# Real-World Data of Anticoagulant Treatment in Non-valvular Atrial Fibrillation

**DOI:** 10.3389/fcvm.2021.733300

**Published:** 2022-01-21

**Authors:** Jose Miguel Calderon, Fernando Martinez, Javier Diaz, Antonio Fernandez, Inmaculada Sauri, Ruth Uso, Jose Luis Trillo, Sara Vela, Carlos Bea, Josep Redon, Maria Jose Forner

**Affiliations:** ^1^Cardiovascular and Renal Research Group, INCLIVA Research Institute, University of Valencia, Valencia, Spain; ^2^Internal Medicine Hospital Clínico de Valencia, Valencia, Spain; ^3^CIBERObn Carlos III Institute, Madrid, Spain

**Keywords:** atrial fibrillation, anticoagulant therapy, stroke, mortality, VKA, NOACs, antiplatelet

## Abstract

**Aims:**

To assess the impact of anticoagulant treatment on risk for stroke and all-cause mortality of patients with atrial fibrillation using real-world data (RWD).

**Methods:**

Patients with prevalent or incident atrial fibrillation were selected throughout a study period of 5 years. Stroke, transitory ischemic attack, hemorrhagic stroke, and all-cause mortality were identified in the claims of the electronic health records (EHRs). Subjects were classified according to the anticoagulant treatment in four groups: untreated, vitamin K antagonists (VKAs), New Oral Anticoagulants (NOACs), and antiplatelet (AP). Risk of events and protection with anticoagulant therapy were calculated by Cox proportional hazard models adjusted by potential confounders.

**Results:**

From a total population of 3,799,884 patients older than 18,123,227 patients with incident or prevalent atrial fibrillation (AF) were identified (mean age 75.2 ± 11.5 years old; 51.9% women). In a follow-up average of 3.2 years, 17,113 patients suffered from an ischemic stroke and transitory ischemic attack (TIA), 780 hemorrhagic stroke, and 42,558 all-cause death (incidence of 46, 8, 2, and 120 per 1,000 patients/year, respectively). Among CHA2DS2, VASc Score equal or >2, 11.7% of patients did not receive any anticoagulant therapy, and a large proportion of patients, 47%, shifted from one treatment to another. Although all kinds of anticoagulant treatments were significantly protective against the events and mortality, NOAC treatment offered significantly better protection compared to the other groups.

**Conclusion:**

In the real world, the use of anticoagulant treatments is far from guidelines recommendations and is characterized by variability in their use. NOACs offered better protection compared with VKAs.

## Introduction

Atrial fibrillation (AF) is the most common sustained arrhythmia in adults being frequent in the aged population (1). As a consequence of multiple conditions (2), it is frequently a marker of underlying cardiac or vascular disease. Associated with the risk of stroke, heart failure, dementia, and mortality (3), it requires treatment to restore sinus rhythm, control heart rate, and reduce embolism by using antiplatelet (AP) or anticoagulant therapy (4, 5). Guidelines in anticoagulant treatment of AF (6–8) have established recommendations about when to start, drugs to be used, dose, and monitoring. However, the efficacy of treatments depends not only on the prescription but also on the maintenance of treatment over time. One of the problems with long-term therapy is the lack of adherence, with frequent discontinuation and changes of medication which impact real life. Anticoagulant treatment is not an exception for the lack of adherence risk, and it is one of the most common treatments, vitamin K antagonists (VKAs), that require frequent monitoring, therefore, reducing treatment adherence.

Although randomized trials provide evidence on the efficacy of anticoagulant treatments, the results may differ from those observed in daily clinical practice. Using real-world data (RWD), the impact of anticoagulant therapy has been analyzed using different sources (9–15). Electronic health records (EHRs) collect a large amount of information, including data about prevalence, disease control, risk factors, treatments, modifying conditions, and information for surveillance programs (16, 17). All of these can aid in evaluating treatment performance outside of the controlled environment of clinical trials.

The objective of the present study was to assess the impact of anticoagulant and AP treatment, in the risk for stroke and all-cause mortality of patients with non-valvular AF using data from the EHR of a general population. Besides the impact of VKA and New Oral Anticoagulants (NOACs), the role of AP drugs was also analyzed.

## Subjects and Methods

### Study Population and Baseline Data Collection

The study population was recruited from the healthcare system of the Valencia Community with a population of 3,799,885 people older than 24 years in 2012. One unique electronic centralized clinical record per patient exists. Total population data were extracted for the period of time between January 1, 2012, and December 31, 2016. Registry included patient demographics, medications, vital status, medical history, diagnostic codes (ICD-9), and laboratory data. Patients' data had a process of pseudo-anonymization, first when the data came to the researchers and second when anonymization was done by masking or deleting variables with risk to re-identify patients. Spanish Law 3/2018 of Data Protection and Guaranty of Digital Rights and corresponding European norms (18) were followed. Committee for Ethics and Clinical Trials of the Hospital Clinic of Valencia approved the study.

Subjects were included in the study from January 1, 2012, if they had an AF diagnosis (ICD-9 427.31 and V07.39O; ICD-10 I48.1, I48.2, and I48.91) before this time. Incident cases of AF during the study period until December 31, 2016, were also included. Patient data were collected from the primary care physician's office, specialists, nurses' offices, pharmacies, hospitals, and emergency departments. As a result, not all the baseline variables that were needed to adjust for potential confounding were available at the exact time of inclusion. Therefore, we defined 6-month windows around the time of study inclusion in order to gather complete information.

### Cardiovascular Risk Factors Definition

Hypertension was defined as an office mean systolic blood pressure ≥140 mm Hg, a mean diastolic blood pressure ≥90 mm Hg, a recorded physician diagnosis, or medication use. Diabetes was defined as non-fasting glucose ≥200 mg/dl, a recorded physician diagnosis, medication use, or an HbA1c **≥**6.5%. Serum total cholesterol was measured enzymatically using the Cholesterol High Performance reagent (Roche Diagnostics, Basel, Switzerland). High-density lipoprotein (HDL) cholesterol was measured using a direct HDL reagent (Roche Diagnostics). Low-density lipoprotein (LDL) cholesterol was calculated by using the Friedewald formula. Dyslipidemia was defined by total cholesterol >200 mg/dl and/or treatment with lipid-lowering drugs. CHA2DS2-VASc (19) was calculated for each patient.

### Cardiovascular Events Definition

Incidences of stroke, transitory ischemic attack, hemorrhagic stroke, and all-cause mortality until December 31, 2016, were collected. Events were assigned from the ICD codes recorded at discharge from hospitalizations or the emergency room. Death was extracted from the death registry. Follow-up was calculated as the difference between the inclusion date and the date of the event, death, or December 31, 2016, whichever occurred first.

### Anticoagulant Treatment

Treatment was collected from the prescription repository of the EHR, with the ATC codes. The initial treatment was the one prescribed at the time of starting the observational period, and the last treatment was the current prescription when the event occurred or at the end of the observational period. Based on the last prescription, three therapy groups were considered: VKAs B01AA, NOAC B01AE, and AP B01AB. Subjects without any of these prescriptions during the whole follow-up period were considered untreated. For each individual, to evaluate the persistence with treatment, the amount of time with a valid prescription without discontinuation previous to the event or until the end of the follow-up period was calculated.

### Statistical Analysis

The incidence rate of events was expressed as the number of cases/1,000 patients/year. To evaluate the impact of persistence with treatment, incidence rates were calculated separately by tertiles of persistence. Incidence of events and categorical variables among groups were compared using the chi-square test. The chi-square test was used to compare the incidence rates across groups of persistence with treatment. Continuous variables among groups were compared using the ANOVA test. The risk of events based on therapy groups was evaluated by means of cumulative survival rates and Cox proportional hazards regression models. Models were adjusted by clinically relevant factors, including age, sex, hypertension, diabetes, coronary heart disease, dyslipidemia, heart failure, CHA2DS2-VASc, and persistence with treatment. The free statistical software R was used for the analysis.

## Results

### General Characteristics of the Study Population

A total of 123,227 patients with prevalent or incident AF were included (mean age 75.2 ± 11.5 years old; 51.9% women, 84.4% hypertensives, 50.7% dyslipidemias, 37.2% diabetics, 21.6 % with HF, and 17.9% with a previous history of coronary heart disease). At the time of inclusion, the average CHA2DS2-VASc Score was 3.46 ± 1.73. The main characteristics of the study population grouped by treatment group at the end of the study are shown in Table 1. The patient/time of observation was a total of 4,896,943 patient-month. Ten subjects per 1,000 patients/year required hospital admission due to gastrointestinal hemorrhage, but no differences among the treatment groups were observed (data not shown).

**Table 1:** Study population grouped by anticoagulant treatment at the end of the study.

**Variable/population**	**TOTAL (123,227)**	**NOAC (19,144)**	**AP (28,257)**	**VKA (56,348)**	**No treatment (19478)**
Age (yr)	75.22 ± 11.47	73.42 ± 10.25	77.75 ± 11.58	75.54 ± 9.33	72.4 ± 16.19
Sex (males)	60,542 (49.1)	9,302 (48.6)	13,259 (46.9)	28,381 (50.4)	9,600 (49.3)
Score chad	3.46 ± 1.73	3.23 ± 1.64	3.8 ± 1.79	3.59 ± 1.59	2.86 ± 1.92
Diabetes	45,897 (37.2)	6,640 (34.7)	11,572 (41.0)	22,271 (39.5)	5,414 (27.8)
Hypertension	103,963 (84.4)	16,497 (86.2)	24,479 (86.6)	49,641 (88.1)	13,346 (68.5)
Dislipidemia	62,438 (50.7)	11,003 (57.5)	14,345 (50.8)	30,503 (54.1)	6,587 (33.8)
Heart failure	26,640 (21.6)	2,727 (14.2)	7,086 (25.1)	13,731 (24.4)	3,096 (15.9)
CHD	22,103 (17.9)	3,101 (16.2)	8,004 (28.3)	9,598 (17.0)	1,400 (7.2)
**Events**					
Stroke and TIA	17,113 (13.9)	3,221 (16.8)	5,370 (19.0)	7,487 (13.3)	1,035 (5.3)
Hemorragic stroke	780 (0.6)	162 (0.8)	179 (0.6)	378 (0.7)	61 (0.3)
All-cause mortality	42,558 (34.5)	2,956 (15.4)	13,859 (49)	16,230 (28.8)	9,513 (48.8)

*Variables are expressed as mean ± SD or as absolute number and percentage ().*

*CHD, coronary heart disease; TIA, Transitory ischemic attack*.

### Anticoagulant Treatment

**Figure 1:**
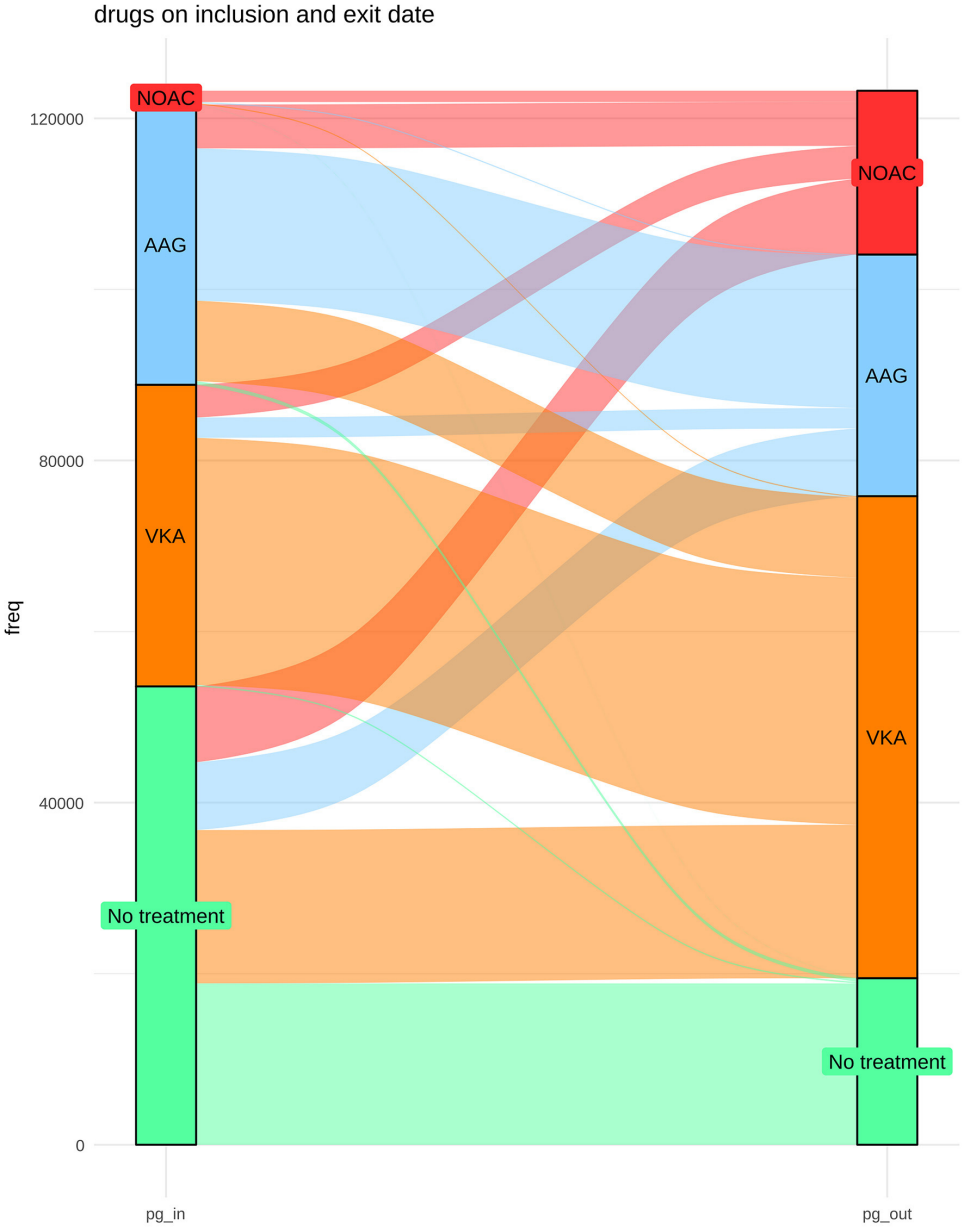
Distribution of treatments at the beginning and at the end of the study period and the changes over time.

**Figure 2:**
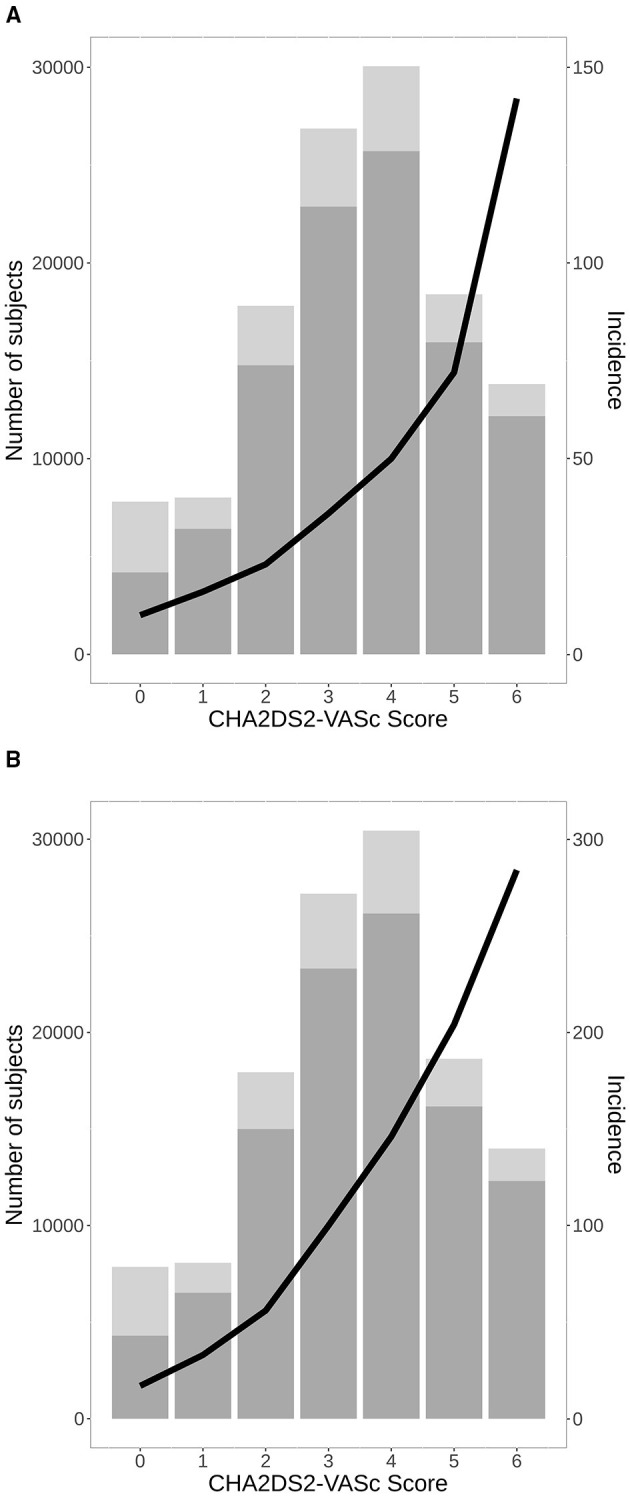
A number of total patients with AF with (bottom) and without (upper) anticoagulant treatment or antiplatelet (AP) in the study period in each category of CHA2DS2-VASc Score, and incidence of stroke per 1,000 patients/year **(A)**, all-cause mortality **(B)**. AF, atrial fibrillation.

The distribution of treatments during the observational period is shown in Figure 1. At the end of the study, the groups were as follows: 56,348 (45.7%) VKA, 19,144 (15.5%) NOAC, and 28,257 (22.9%) AP. The list of drugs of each class is shown in Table 2. Untreated during the study period were 19,478 patients (15.8%). The number of patients treated and untreated in each category of the CHA2DS2-VASc Score is shown in Figure 2. Selecting those with the CHA2DS2-VASc Scores equal to or greater than two, 14,421 (11.7%) subjects neither received anticoagulant nor antiaggregant therapy. The total time with a valid prescription for each group was 1,933,188 person-months in the AVK, 393,770 person-months in NOAC, and 754,667 person-months in the AP group.

**Table 2:** Pharmacologic compounds in the study population.

**Treatment**	**Number (%)**
**NOAC**	
Davigatran	5,367 (5.2)
Rivaroxaban	6,454 (6.2)
Apixaban	6,985 (6.7)
Edoxaban	338 (0.3)
**VKA**	
Acenocumarol	54,829 (52.8)
Warfarin	1,519 (2.7)
**AP**	
Acetilsalicilic acid	22,718 (21.9)
Clopidogrel + Acetilsalicilic acid	232 (0.2)
Clopidogrel	4,328 (4.2)
Prasugrel	33 (0)
Ticagrelor	70 (0.1)
Dipiridamol	38 (0)
Triflusal	810 (0.1)
Ticlopidin	28 (0)

*Absolute number and percentage (). VKAs, vitamin K antagonists; NOACs, New Oral Anticoagulants; AP, antiplatelet*.

### Stroke Incidence

**Figure 3:**
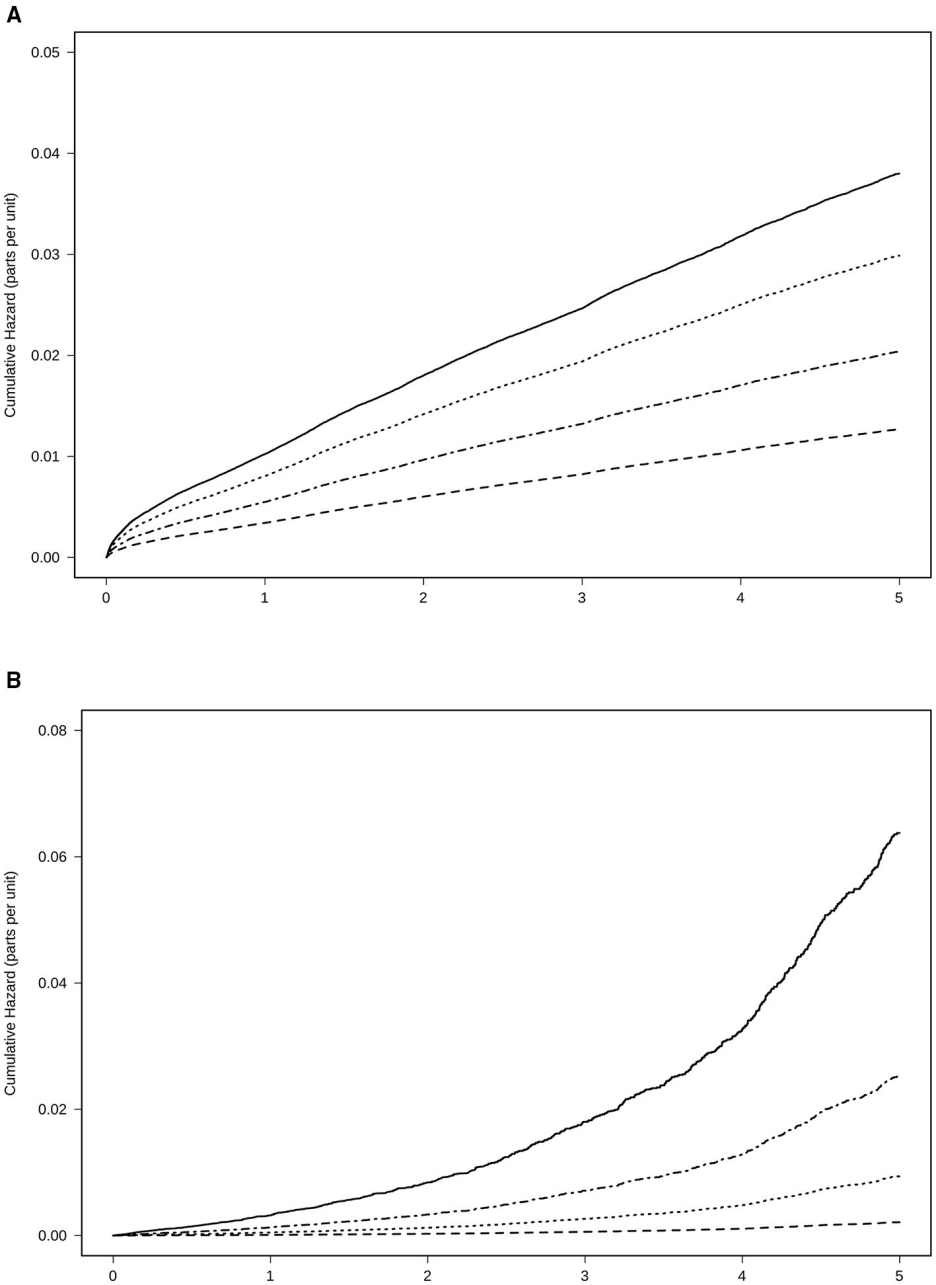
Risk of events for anticoagulant treatment adjusted by age, sex, CHA2DS2-VASc Score, hypertension, diabetes, dyslipidemia, coronary heart disease, heart failure, and time in the persistence of each kind of treatment. **(A)** Stroke and transitory ischemic attack. **(B)** Haemorrhagic stroke. Treatment with NOAC 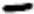, VKA 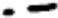, AP 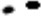, No treatment 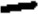 VKAs, vitamin K antagonists; NOACs, New Oral Anticoagulants; AP, antiplatelet.

A total of 15,819 patients suffered from a stroke and 2,849 patients from transitory ischemic attack (TIA). Overall, the incidence of stroke and TIA were 46 and 8 events per 1,000 patients/year, respectively. In patients with at least 6 months of therapy, the three groups of treatment had a reduced risk of stroke and TIA as compared to the absence of treatment, adjusted by age, sex, the CHA2DS2-VASc Score, hypertension, diabetes, dyslipidemia, coronary heart disease, heart failure, and months persistent with treatment. NACOs achieved the maximal protection of stroke and TIA [0.33 (0.31–0.36)], followed by VKA [0.54 (0.51–0.56)] and the AP [0.79 (0.75–0.83)], Figure 3A. Hemorrhagic stroke was diagnosed in 780 subjects, with an incidence of 2 hemorrhagic events per 1,000 patients/year. The risk not being significantly higher in the VKA [1.12 (0.87–1.44)] as compared to the other treatment groups, NOAC [0.46 (0.33–0.64)], and AP [0.68 (0.51–0.90)], Figure 3B. The risk reduction, HR, for each treatment and the statistical significance among therapeutic groups are shown in Table 3. In all groups, there was a significant trend in the incident of events regarding the amount of time with a valid prescription, the more time a patient was on a prescription the less incidence of events (*p* < 0.01), Table 4.

**Table 3:** Reduction of risk of each kind of anticoagulant treatment.

	**Stroke and TIA**	**Haemorrhagic**	**All, cause mortality**
		**stroke**	
No treatment	1	1	1
NOAC	0.33 (0.31–0.36)*^&^	0.46 (0.33–0.64)*	0.20 (0.19–0.21)*^&^
AP	0.79 (0.75–0.83)*	0.68 (0.51–0.90)*	0.46 (0.45–0.47)*
VKA	0.54 (0.51–0.56)*^$^	1.12 (0.87–1.44)	0.29 (0.28–0.30)*^$^

*p values against No treatment < 0.001.

*^&^p values against VKA and AP < 0.01.*

*^$^p-values against AP < 0.05.*

*VKAs, vitamin K antagonists; NOACs, New Oral Anticoagulants; AP, antiplatelet*.

**Table 4:** Incidence of neurologic events and all-cause mortality considering the time of use of anticoagulant treatment.

**Persistent treatment (months)**		**NOAC**	**AP**	**VKA**
**Stroke plus TIA**				
Between 20 and 40	Incidence Number events Number patients	12 240 4,982	55 1,191 6,576	48 2,269 14,413
> 40	Incidence Number events Number patients	6*** 72 2,398	10*** 383 7,653	9*** 1,063 23,010
**Haemorrhagic stroke**				
Between 20 and 40	Incidence Number events Number patients	1 21 5,780	1 28 6,837	4 182 14,383
> 40	Incidence Number events Number patients	0* 3 2,761	1 31 8,567	1*** 155 25,249
**All-cause mortality**				
Between 20 and 40	Incidence Number events Number patients	29 662 5,804	149 3,424 6,852	111 5,336 14,293
>40	Incidence Number events Number patients	11*** 150 2,768	33*** 1,359 8,578	23*** 2,873 25,243

*Incidence rate per 1,000 patients/year.*

Chi-2 *p < 0.05; ***p < 0.001.

### Mortality

**Figure 4:**
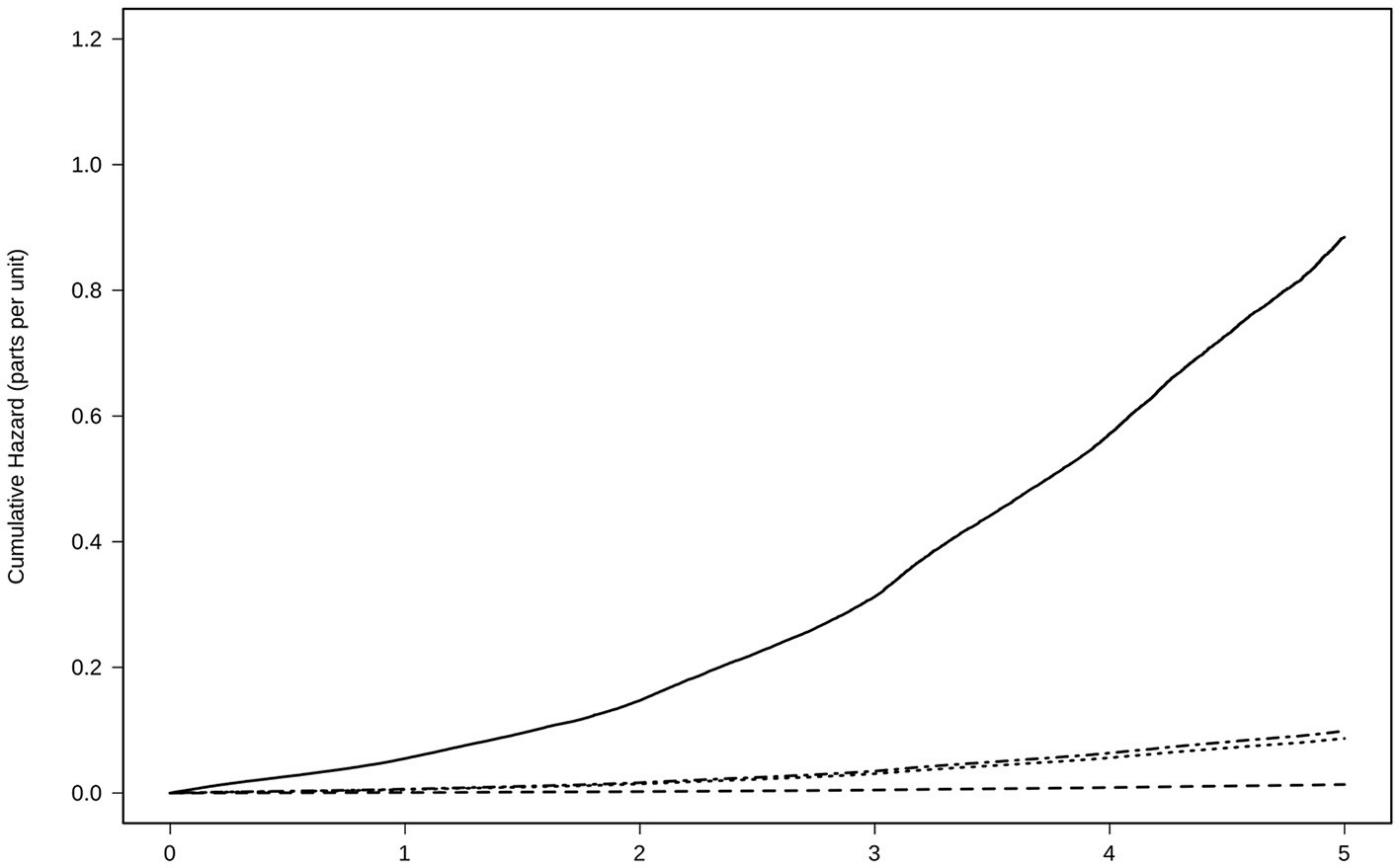
Risk of all-cause mortality for anticoagulant treatment adjusted by age, sex, CHA2DS2-VASc Score, hypertension, diabetes, dyslipidemia, coronary heart disease, heart failure, and time in the persistence of each kind of treatment. Treatment with NOAC 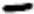, VKA 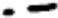, AP 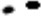, No treatment 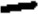 VKAs, vitamin K antagonists; NOACs, New Oral Anticoagulants; AP, antiplatelet.

The number of deaths was 42,558 (incidence of all-cause mortality, 120 per 1,000 subjects/year). All groups with treatment were able to reduce the risk of death by any cause compared to the absence of treatment. In the fully adjusted Cox regression model, the lowest risk was achieved using NOACs [0.20 (0.19–0.21)], followed by AVK [0.29 (0.28–0.30)], and AP [0.46 (0.45–0.47)], Table 2 and Figure 4.

## Discussion

In the real-world setting, large variability in the use of anticoagulants for non-valvular AF was observed. In total, 11.7% of patients with a CHA2DS2-VASc Score equal to or greater than 2 did not receive any anticoagulant therapy during the study period, 22.9% received AP drugs, and a large proportion of patients, 47%, shifted from one treatment to others. Although all the anticoagulants and even the AP treatments reduce the risk of stroke and all-cause mortality, the NOACs seem to offer significantly better protection than the VKAs without more hemorrhagic events. Risk reduction with AP was inferior to the one obtained with anticoagulants.

Although clinical trials are the gold standard to assess the efficacy and safety of a drug, RWD data may be used to reflect the broader picture of various clinical settings, provide supplementary insight, and identify gaps (10). RWD studies could help evaluate the effectiveness of healthcare interventions (15) and cost-effectiveness studies, helping policy-makers to make decisions on the optimal allocation of resources (20). The present study was conducted with EHRs associated with a public general practice setting that covers 92% coverage of the population living in the area. The EHR guarantees the interoperability of all sources of information which included all diagnostics, risk factors, prescriptions, hospitalization, and mortality linked to the same code. The present study addressed the impact of the efficacy and safety of anticoagulant drugs in non-valvular AF in real-world use of drugs and shifts on treatment.

Several studies and meta-analyses (14, 15) have described aspects of the anticoagulation of AF with RWD data, although the results sometimes are misleading due to the lack of uniformity of the studies selected in the meta-analysis. The majority compared the performance of the NOACs with the VKA in terms of risk reduction of stroke or major bleeding events (9.11, 12, 14, 16), with or without applying propensity score matching (9). Our results emphasize the importance of anticoagulation to reduce stroke incidence, cardiovascular, and total mortality. The NOACs were more effective and safer than the other groups, and the persistence with treatment increased the protection.

Less attention had been paid to treatment adherence, inertia, and the use of AP drugs. One study observed suboptimal adherence and persistence to NOACs in patients with AF, with one in three patients adhering to their NOAC <80% of the time. Moreover, the lack of adherence was associated with poor clinical outcomes (13) as was observed in the present study. Also, of concern is that a significant proportion of therapeutic inertia, high-risk patients for stroke were not receiving guideline-recommended therapy (21). The reasons for this under treatment are multifactorial and include patient and physician-driven factors (22). The AP group merits further comment. A large number of subjects are receiving AP drugs for different reasons, among them, frail patients are those with post-coronary stenting. What is relevant in the present data is that AP drugs also reduce the risk of stroke, hemorrhagic stroke, and mortality although protection is lower as compared with anticoagulants.

Although right now this may be different, acenocoumarol was the most frequently prescribed anticoagulant treatment in our patients, with the same being true in other parts of Spain (23). As the expected trend to shift to NOACs was observed during the follow-up.

The strengths and limitations of the study should be discussed. The EHR of the present study covers 92% of the population in our community and guarantees the interoperability of all sources of information, including claim data, diagnoses, risk factors, prescriptions, hospitalizations, and mortality records linked to the same code. We included prevalent AF and incident cases to have a broader picture of the risk associated with AF in our community. A large number of patients with AF were selected with a relatively long follow-up accounting for potential confounders, such as age, sex, and major cardiovascular risk factors. Moreover, treatment persistence was also considered as a measure of adherence. Some limitations, such as the presence of a high percentage of missing values, are inherent to the EHR. To minimize its impact, only patients with the required variables for the analysis were selected. Some important variables, such as smoking, were not reliable enough to be included as co-variables.

As events were adjudicated from the ICD codes, some misclassification was possible. However, the large sample size makes the potential impact of misclassification minimal. The reasons for the lack of treatment, the quality of VKA control, and the dosage or type of NOACs were not analyzed. Regarding stroke, all types were included, and not only the cardio-embolic type, which is what may partly explain the results observed for the AP group. Also, cardiovascular deaths were not considered separately, but it is expected that the results would be similar. Finally, we did not gather information about the reasons for shifts from one treatment to another.

The 2020 ESC guideline on diagnosis and management of AF has recently upgraded treatment recommendations for switching from VKA to NOAC. It is now recommended and indicated for patients on VKA who have a time in the therapeutic range below 70%. Overall, the primary goal is to increase the percentage of treated patients and their adherence to the treatment.

In conclusion, the present study demonstrated that real-life anticoagulant treatment is far from the guidelines' recommendations. A high proportion of high-risk untreated subjects was present, and shifts of treatments from one class to another were frequent. Although potential advantages of NOACs compared to VKA were present, the prescription rate of anticoagulants should be increased to reduce mortality and the incidence of neurologic events.

## Data Availability Statement

The datasets presented in this article are not readily available because data are poblational and pertain to Health Care Authorities. Requests to access the datasets should be directed to Josep Redon, josep.redon@uv.es.

## Ethics Statement

The studies involving human participants were reviewed and approved by Committee for Ethics and Clinical Trials of the Hospital Clinico of Valencia. Written informed consent for participation was not required for this study in accordance with the National Legislation and the Institutional requirements.

## Author Contributions

JC, FM, and JR: conceptualization. FM, JR, and MF: methodology. AF, IS, JD, RU, and JT: formal analysis. SV, CB, JR, and MF: investigation. AF, IS, and JC: data curation. JC, MF, and JR: writing—original draft preparation. MF, JR, JD, RU, and JT: writing—review and editing. All authors have read and agreed to the published version of the manuscript.

## Funding

The authors declare that this study received funding from BigData@heart (IMI2, FPP116074,2); BIGMEDILYTICS (ICT,15, 780495); and PI16/01402, CIBEROBN Institute of Health Carlos III. The funder was not involved in the study design, collection, analysis, interpretation of data, the writing of this article or the decision to submit it for publication.

## Conflict of Interest

The authors declare that the research was conducted in the absence of any commercial or financial relationships that could be construed as a potential conflict of interest.

## Publisher's Note

All claims expressed in this article are solely those of the authors and do not necessarily represent those of their affiliated organizations, or those of the publisher, the editors and the reviewers. Any product that may be evaluated in this article, or claim that may be made by its manufacturer, is not guaranteed or endorsed by the publisher.
